# Inhibitory control and counterintuitive science and maths reasoning in adolescence

**DOI:** 10.1371/journal.pone.0198973

**Published:** 2018-06-21

**Authors:** Annie Brookman-Byrne, Denis Mareschal, Andrew K. Tolmie, Iroise Dumontheil

**Affiliations:** 1 Centre for Brain and Cognitive Development, Department of Psychological Sciences, Birkbeck, University of London, London, United Kingdom; 2 Centre for Educational Neuroscience, University of London, London, United Kingdom; 3 Department of Psychology and Human Development, UCL Institute of Education, University College London, London, United Kingdom; Katholieke Universiteit Leuven, BELGIUM

## Abstract

Existing concepts can be a major barrier to learning new counterintuitive concepts that contradict pre-existing experience-based beliefs or misleading perceptual cues. When reasoning about counterintuitive concepts, inhibitory control is thought to enable the suppression of incorrect concepts. This study investigated the association between inhibitory control and counterintuitive science and maths reasoning in adolescents (*N* = 90, 11–15 years). Both response and semantic inhibition were associated with counterintuitive science and maths reasoning, when controlling for age, general cognitive ability, and performance in control science and maths trials. Better response inhibition was associated with longer reaction times in counterintuitive trials, while better semantic inhibition was associated with higher accuracy in counterintuitive trials. This novel finding suggests that different aspects of inhibitory control may offer unique contributions to counterintuitive reasoning during adolescence and provides further support for the hypothesis that inhibitory control plays a role in science and maths reasoning.

## Introduction

**Fig 1:**
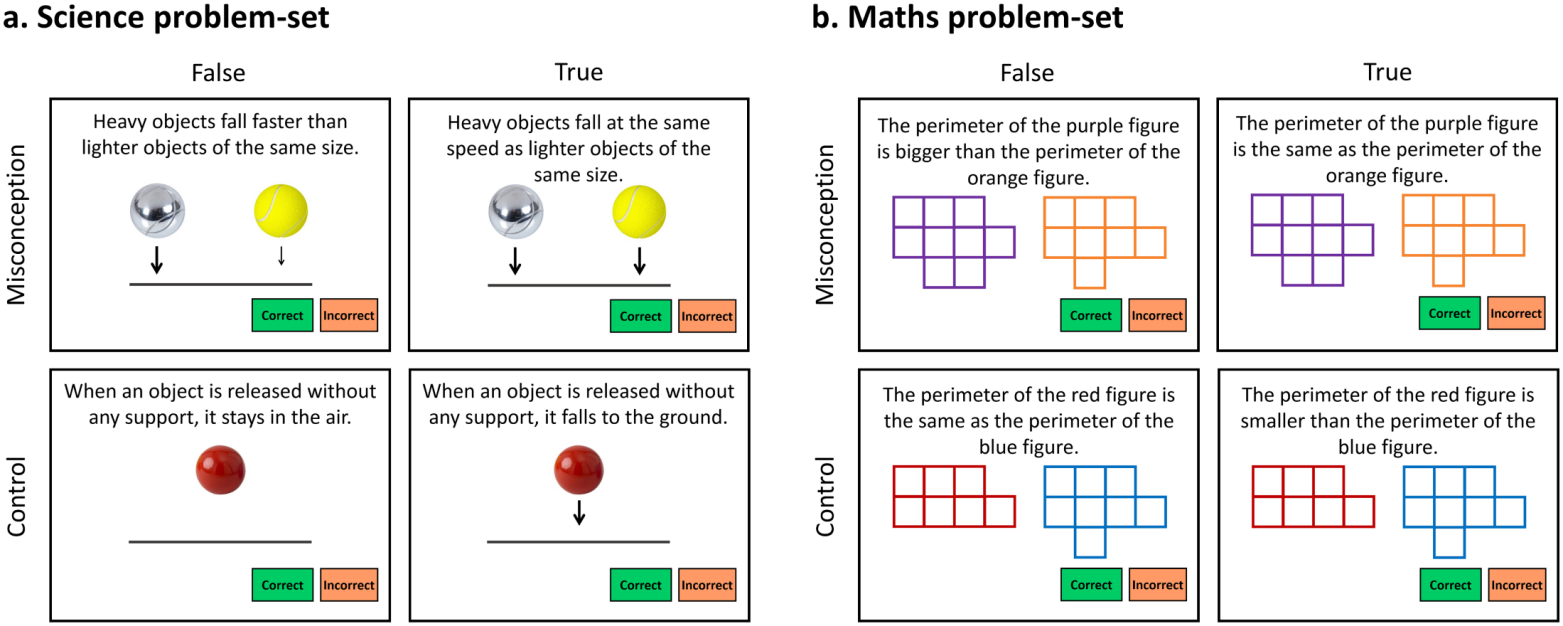
Example problem-sets for (a) science and (b) maths. Text and image size has been increased to enhance legibility. Correct and incorrect ‘buttons’ remained on the screen to remind participants which key to press.

The acquisition of abstract concepts reflecting an understanding of how elements in the world relate to one another underpins school-based learning of science and maths [1]. These abstract concepts go beyond what is immediately perceptually available, and sometimes go against prior experience, beliefs or perceptual evidence. Learning new concepts is therefore constrained by pupils’ ability to overcome conflicting information. Conceptual change, the process of acquiring a new explanatory framework for a certain phenomenon, has been argued to be a key challenge faced by science educators [2], requiring more than learning new facts and going beyond the enrichment of previously held notions [3]. Similarly, in maths, when learning and applying new concepts, pupils can be misled by prior beliefs, generalisation of previous learning (e.g. in the case where for integers 5 > 2 while for fractions 1/5 < 1/2) [4] or perceptual evidence (e.g. when a larger surface area may not be associated with a larger perimeter, see Fig 1b) [5–7]. While students used to be thought to learn new concepts through the replacement, reorganisation, or restructuring of previously held concepts [3,8], newer research suggests that prior beliefs remain in the face of new evidence and can lead to science and maths misconceptions that are notoriously resistant to change, persisting throughout school education and well into adulthood [1,6,9,10]. For example, a study with adults educated in science found that accuracy was lower and response times longer in statements where naïve and scientific theories were inconsistent (i.e. counterintuitive), compared to statements consistent across naïve and scientific theories (i.e. intuitive) [11]. This is evidence of an ongoing requirement to suppress intuitive responses even when the correct answer has been learnt.

Inhibitory control, the ability to suppress a prepotent response or irrelevant information, is thought to allow the suppression of naïve theories, incorrect strategies, or misleading perceptual cues during counterintuitive reasoning in science and maths [1,12–17]. A better understanding of the learning of science and maths is particularly important, as these disciplines are of enormous economic impact, are compulsory subjects in primary and secondary school, and are considered particularly difficult subjects to learn [18,19].

Most previous research exploring the cognitive processes behind science and maths misconceptions has been performed on children [12,16,19–21] or adults [13,14,17]. The current study investigated the role of inhibitory control in counterintuitive reasoning, i.e. reasoning about concepts where a misconception may be held, during adolescence, a period when inhibitory control continues to develop [22] and individuals learn increasingly advanced concepts in both science and maths [23,24].

Inhibitory control has been classified in various ways. Nigg [25], for example, outlined eight kinds of inhibition, while others tend to group inhibitory control into two categories [26]. Behavioural, or response inhibition, is the suppression of a motor response, whereas interference control, or semantic inhibition, is the suppression of meaning in the face of conflict. In a simple Go/No-Go response inhibition task, rapid frequent responses are made to Go stimuli, with infrequent non-responses to No-Go stimuli [27]. Errors of commission occur when the dominant Go response has not been successfully inhibited in a No-Go trial. In a complex version, No-Go events are determined by both the current and previous trial [27]. As the complex Go/No-Go requires participants to keep the current and previous trial in mind to perform accurately, it provides a measure of inhibitory control in the context of a cognitive load. In a Stroop semantic inhibition task, conflicting information is presented simultaneously and the less salient aspect of the stimulus is responded to while the dominant aspect is inhibited [28]. The Stroop therefore requires the suppression of one type of meaning alongside the processing of another. Performance on the simple Go/No-Go [29,30], complex Go/No-Go [29], and Stroop [22,31] tends to improve through adolescence. Attempts to establish the extent of overlap between response and semantic inhibition have yielded mixed results [32,33], indicating that measures of inhibition are influenced by different underlying mechanisms [34], but are not totally independent [26]. With this in mind, the current study measured both response and semantic inhibition, in order to establish the individual roles that each might play in counterintuitive reasoning.

The link between science and maths reasoning in general, or counterintuitive reasoning specifically, and inhibitory control has been examined across development using correlational, priming, and neuroimaging studies. Evidence from correlational studies suggests that children with better inhibitory control perform better on science problems requiring counterintuitive reasoning. In the domain of physics, the tubes task had an experimenter drop a ball down one of three opaque tubes that crossed over [35]. Three- and four-year-olds were asked to point to the opaque container where the ball landed. The most common error was to choose the container directly beneath the opening of the tube, suggesting that the toddlers’ gravity theory (that the ball would fall in a straight trajectory) was not successfully inhibited in favour of an object solidity theory (that the ball would follow the solid tube). Since looking time paradigms reveal an understanding of object solidity at this age [36], the authors argued that the task engaged a number of strategies, and that selection of the correct strategy depended on the inhibition of incorrect strategies. This interpretation was further supported by a positive correlation between tubes task performance and inhibitory control as measured by a gift delay task [35]. In biology, mature biological understandings of life, death, and bodily functions were predicted in five- to seven-year-olds by an aggregate measure of executive function that reflected performance on tasks requiring a combination of shifting, working memory, and either response inhibition or semantic inhibition [19]. Although the executive function measure was not purely inhibitory control, the authors suggested that inhibitory control is one skill that enabled the suppression of naïve biological theories when a mature conceptual understanding was shown. The picture is, however, inconsistent, as inhibitory control was not related to science performance in other studies in 11-year-olds [37] or 12- to 13-year-olds [38,39].

In the domain of maths, overlapping strategy use in problem solving is a particular demonstration of the maintenance of multiple concepts and theories over the course of learning, rather than the replacement of old concepts by new concepts. An examination of strategy use in four- and five-year-olds solving maths sums found that even when new, more sophisticated strategies had been learnt, children continued to use old strategies [40,41]. The concurrent existence of multiple strategies suggests that inhibitory control is likely to be involved from a young age to allow selection of the best strategy through suppression of the alternatives. When inhibitory control was measured directly through a Stroop task, performance in three- to six-year-olds was associated with scores on a standardised maths test in one experiment, and with magnitude comparison in another [21]. Similarly, a study of 11- to 14-year-olds found that maths achievement was correlated with both numerical and non-numerical semantic inhibitory control [20]. A further maths study investigated inhibitory control in 14-year-olds who were instructed to use a new strategy for solving algebra word problems [42]. Better response and semantic inhibitory control predicted higher accuracy as well as fewer intrusions of the previous strategy [42], indicating that inhibitory control suppressed the previous strategy in effective maths reasoning. As with science, inhibitory control does not always relate to school performance, as evidenced by one study where 5- to 6-year-olds’ inhibition did not predict maths achievement [43]. A meta-analysis showed a modest overall association between inhibitory control and maths in young children [44]. These studies investigated overall associations between inhibitory control and general maths performance, but did not specifically focus on counterintuitive reasoning.

A few studies have used priming to probe the role of inhibitory control in counterintuitive maths reasoning. Nine-year-olds performed better on a counterintuitive number conservation trial if they were primed to inhibit through the successful inhibition of an incongruent Stroop trial [45]. Ten-year-olds performed better on a number conservation or class inclusion task when primed by a trial from the other task requiring the inhibition of a misleading strategy [12]. In a further study spanning three age groups, children (~ 12 years old), adolescents (~ 15 years old) and adults showed slower response times when a probe problem with congruent relational term and arithmetic operation (“more than” > addition) followed a prime problem with incongruent relational term and arithmetic operation (“more than” > subtraction). This negative priming was interpreted as reflecting that successfully solving these arithmetic counterintuitive problems required the inhibition of an incorrect strategy [16].

Neuroimaging work on logical and scientific reasoning in adults has consistently shown that the inhibition of pre-existing beliefs, misleading perceptual-biases, and intuitive heuristics during counterintuitive reasoning is associated with the activation of the anterior cingulate cortex and the prefrontal cortex, notably the inferior frontal cortex and dorsolateral prefrontal cortex, thought to reflect the recruitment of greater cognitive control for counterintutive vs. intuitive reasoning [12,14,17,46–49]. Further consistent evidence comes from Houdé and colleagues [50], who attempted to shift adult’s responses from a perceptual bias to logical reasoning in a rule falsification task and showed that this shift was associated with increased recruitment of prefrontal, versus posterior, brain regions, which was interpreted as reflecting their inhibition of a misleading strategy.

The research summarised above suggests that naïve theories, prior knowledge, and misleading perceptual cues are inhibited during successful science and maths reasoning. Adolescents are faced with increasingly complex, and sometimes counterintuitive, science and maths concepts through compulsory school curricula, while their inhibitory control abilities are still developing. The current study aimed to investigate the association between inhibitory control and counterintuitive science and maths reasoning in 11- to 15-year-olds.

A novel science and maths misconceptions task was designed to measure adolescents’ ability to give the correct (although counterintuitive) answer when faced with problems known to be associated with misconceptions. Only misconceptions relevant to the curriculum for 11- to 14-year-olds (Key Stage 3 for England) were included, based on consultation with curricula [23,24] and student study guides [51,52] to ensure validity. While previous studies focused on just one or two misconceptions [13,17,49], the aim here was to cover a wide range of topics to increase the relevance of our findings to education. Control science and maths problems that did not require counterintuitive reasoning (but were related to the counterintuitive concepts) were also included, to account for discipline-specific factors such as knowledge and interest in science and maths, however our predictions and analyses focused on the importance of inhibitory control for counterintuitive reasoning specifically, rather than for science and maths achievement in general (e.g. [20]). The youngest participants were at the start of the Key Stage 3 curriculum in England (and the start of secondary school), and the oldest had completed the Key Stage 3 curriculum, allowing the inclusion of the same problems for all participants.

Both response and semantic inhibition were measured to investigate the possible unique influence of these aspects of inhibitory control. Response inhibition was measured using a simple and complex Go/No-Go adapted from Watanabe et al. [53], to investigate the possibility that response inhibition in the context of higher cognitive load, namely a 1-back working memory load, would be more associated with complex science and maths reasoning, where information may need to be maintained and manipulated while the answer is worked out, compared to inhibitory control within a simpler task. Previous research has shown that simple and complex Go/No-Go tasks are associated with different brain networks [27]. Semantic inhibition was measured with a numerical Stroop adapted from Khng and Lee [34].

It was hypothesised that better semantic inhibitory control, evidenced by less interference effect on accuracy and reaction time (RT) in the numerical Stroop task, would allow participants to better solve the conflict between their naïve belief or misleading perceptual information and the correct answer, and that they would therefore show more accurate and faster responses on the science and maths misconception problems, relative to non-counterintuitive control problems. It was also hypothesised that better response inhibition, evidenced by higher accuracy in simple and complex No-Go trials, would also be associated with better science and maths misconception performance, by limiting impulsive responses. As science and maths problem solving typically requires the maintenance of some information in working memory, and as misconceptions in particular may elicit competition between, and comparison of, intuitive and counterintuitive responses, it was finally hypothesised that performance in complex No-Go trials would show a greater association with science and maths misconception performance than performance in simple No-Go trials.

## Methods

This project received approval from the Department of Psychological Sciences Ethics Committee, Birkbeck College University of London [reference approval number: 141552].

### Participants

Ninety pupils with no neurological or developmental disorders, from an English secondary school where most students are from minority ethnic heritages, and the proportion of free school meals (determined by parental income-related benefits) is well above average, took part (Table 1). Letters were sent to parents of 11- to 15-year-olds (in Years 7 to 10), inviting their children to take part. Written informed parental consent was obtained, where parents confirmed that their children had no neurological or developmental disorders. Participants aged 11 or 12 years verbally consented, while 13- to 15-year-olds provided written consent, in accordance with the guidelines of the local ethics committee, which approved the study.

**Table 1:** Participant characteristics.

		Age (years)		Girls: Boys	WASI Vocabulary		WASI Matrix Reasoning	
					Raw scores	Standardised scores	Raw scores	Standardised scores
Age group	*n*	*M (SD)*	*Range*	*n*	*M (SD)*	*M (SD)*	*M (SD)*	*M (SD)*
12y	25	12.14 (0.30)	11.75–12.67	13:12	33.01 (3.51)	105.82 (8.63)	19.00 (3.46)	103.54 (13.28)
13y	25	13.26 (0.31)	12.75–13.75	17:8	33.12 (3.69)	102.16 (9.87)	18.12 (3.14)	97.45 (10.41)
14y	21	14.32 (0.29)	13.92–14.75	9:12	33.71 (5.02)	100.65 (12.08)	19.67 (2.92)	101.22 (11.14)
15y	19	15.21 (0.35)	14.75–15.75	12:7	35.26 (3.21)	101.43 (7.94)	18.32 (4.06)	95.19 (13.27)

Age groups did not differ in raw Vocabulary scores, *p* = .238, raw Matrix Reasoning scores, *p* = .423, or gender distribution, *p* = .332.

### Tasks

#### Science and maths misconceptions

The science and maths misconceptions task was administered on a laptop. On each trial, participants read a statement relating to science or maths, and pressed one of two keys to indicate whether they thought the statement was correct or incorrect. There were 48 problem-sets, each addressing one curriculum-related misconception where the intuitive response is incorrect, based on research findings (e.g. [6,7,54]).

Each problem-set contained four problems (Fig 1). A *Misconception-False* problem presented a false statement based on a counterintuitive concept, while a *Misconception-True* problem presented a true statement based on the same counterintuitive concept. All misconception problems required counterintuitive reasoning; the intuitive response was incorrect. Varying the nature of the statements was necessary to counterbalance left/right answers across the experiment: Including *Misconception-False* problems only would have led to the correct answer being “Incorrect” on all trials. Note that it was expected that accuracy and RT might be influenced by true or false statement type, since it might be easier to give a correct response when the statement is true.

Knowledge and interest in the topic and task-general factors such as processing speed and attention, were controlled for with problems where no misconception was expected (*Control-False*, *Control-True*) (Fig 1). Efforts were made to ensure that misconception and control problems were matched on statement length, positive versus negative wording, and terminology. It was anticipated that RTs would be faster and accuracy higher in control trials (where counterintuitive reasoning is not required) compared to misconception trials.

Fig 1 presents two examples of problem-sets. The science problem-set (Fig 1a) refers to the misconception that heavy objects fall faster than lighter objects. To correctly solve the *Misconception* problems, participants need to inhibit their naïve belief that the weight of objects matters in this situation. The *Control* problems also refer to the fall of objects, but in this case the concept is that objects fall to the ground if they are not supported. As this is not a misconception participants do not have to inhibit a prior belief. The maths problem-set (Fig 1b) refers to the misconception that a shape with a larger area will also have a larger perimeter. To answer correctly on *Misconception* trials, participants need to inhibit their perception of the larger area of the purple figure compared to the orange figure, which is misleading and irrelevant, and instead focus on assessing the perimeter of the two shapes. The *Control* problems also ask about perimeters but in this case the area and perimeters of the shapes are consistent, therefore there is no misleading perceptual information to inhibit to obtain the correct answer. It was expected that participants with better inhibitory control would be able to answer *Misconception* problems more quickly (as the incorrect belief or irrelevant perceptual information is more quickly inhibited) and accurately (as the correct belief or correct perceptual information is eventually selected) than those with poorer inhibitory control, who might answer intuitively, or take longer to inhibit the incorrect belief or irrelevant perceptual information.

Twenty-six problem-sets were science-based. Biology topics included living organisms, cells, inheritance, genetics, and plants (8 problem-sets). Chemistry topics included pollution, atoms, pure and impure substances, heating, and melting (7 problem-sets). Physics topics included force, the solar system, electricity, gravity, waves, and temperature (11 problem-sets). Maths topics included fractions, decimals, angles, algebra, shape, transformations, statistics, probability, and graphs (22 problem-sets). One hundred and twenty-six problems were accompanied by images (biology: 28/32; chemistry: 18/28; physics: 36/44; maths: 44/88) that were sometimes essential for the problem, sometimes provided further explanation, and sometimes simply relevant to keep the task engaging. In all cases the *Misconception-True/False* and *Control-True/False* problems were matched as well as possible in terms of text and images presented, ensuring all participants saw similar stimuli across conditions, and allowing the comparison of participants’ performance across conditions. See S1 Fig for more example problem-sets.

Two sequences of 96 trials were created by distributing problems such that each sequence contained one misconception trial and one control trial from each problem-set. Two further sequences were created by reversing the presentation order of the original sequences. Each participant thus completed 24 of each of the four problem-set types (*Misconception-False*, *Misconception-True*, *Control-False*, *Control-True*). Participants were assigned to a sequence pseudo-randomly, ensuring each age group contained the range of sequences. Stimuli remained on the screen until a response was made, and the task lasted 11 min on average including self-timed breaks every 24 trials. Accuracy and RT were recorded.

#### Inhibitory control

**Fig 2:**
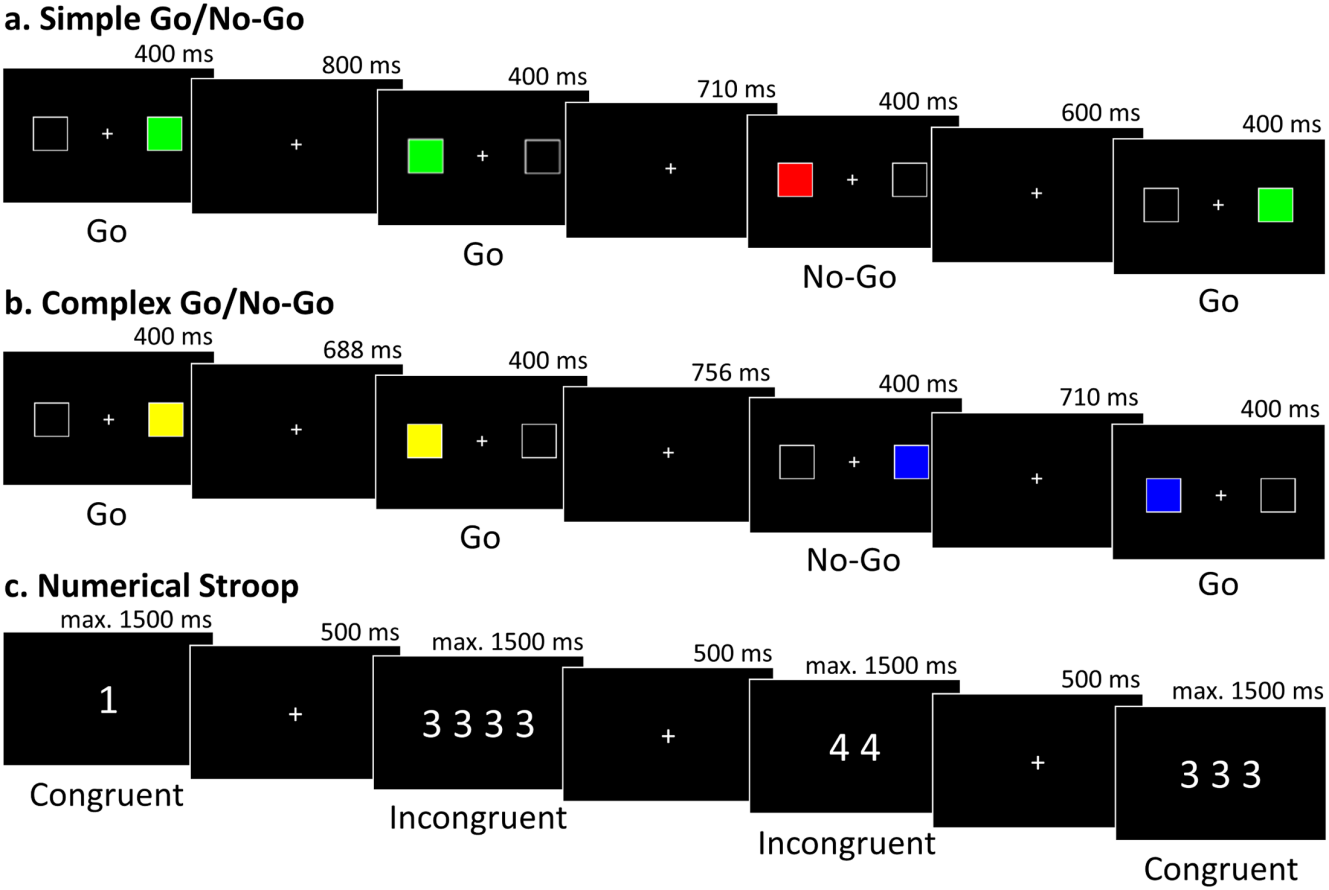
Example time course of events in the inhibitory control tasks. (a) In the simple Go/No-Go, participants pressed the left or right key to indicate the location of the green square (Go trials), but withheld their response when the square was red (No-Go trials). (b) In the complex Go/No-Go, participants pressed the left or right key to indicate the location of the coloured square (Go trials), but withheld their response when a blue square followed a yellow square (No-Go trials). In both tasks 25% of trials were No-Go, as in previous studies (e.g. [29]), so that non-responses were infrequent and thus harder to inhibit, and the inter-stimulus interval was jittered between 600 and 800 ms. (c) In the numerical Stroop, participants pressed the key corresponding to the number of digits on the screen. On congruent trials, the number of digits and the digits themselves matched, while on incongruent trials they differed and participants had to inhibit the representation of the digits. Fifty percent of trials were incongruent as in prior tests of semantic inhibition (e.g. [21]) to maintain high levels of conflict and allow accuracy and RT comparisons between trial types. Stimuli remained on the screen until the participant responded or for a maximum of 1500 ms.

Simple and complex versions of a Go/No-Go task, measuring response inhibition, and a numerical Stroop task, measuring semantic inhibition, were administered on a laptop (Fig 2). See S1 File for full details of these tasks.

#### Wechsler abbreviated scale of intelligence (WASI)

The Vocabulary and Matrix Reasoning subtests of the WASI-II [55] were administered using the stimulus book to control for the contribution of general cognitive ability to science and maths performance (Table 1). The Vocabulary subtest requires participants to explain the meaning of words, while the Matrix Reasoning subtest requires participants to choose a picture that completes a pattern.

### Procedure

Participants were tested in a quiet space in school for approximately 45 min during the school day. The experimenter described each computerised task, emphasising that responses should be given as quickly and accurately as possible. The tasks were performed in the following order: simple Go/No-Go, complex Go/No-Go, numerical Stroop, science and maths misconceptions, WASI Vocabulary, and WASI Matrix Reasoning. Two experimenters collected the data, testing 74 and 16 participants respectively. Participants were given no results and no rewards for taking part, and it was explained that their responses would remain anonymous and independent of school assessments.

### Statistical analysis

Mean RTs are reported for all trials in the science and maths misconceptions task, since RTs for incorrect trials are of interest here, reflecting the time spent to reason about a counterintuitive concept, even if the resulting answer is incorrect. Mean RTs are reported for correct trials only in the inhibitory control tasks (RTs for correct Go trials only in the Go/No-Go). Examination of boxplots across tasks showed outliers, so exclusionary criteria were put in place before analysis commenced. Participants whose mean accuracy or RT was further than ±3.29 standard deviations away from the group mean were excluded from analyses of the task on which they were an outlier, as standardised scores outside that range are cause for concern [56]. Effect sizes are reported as partial eta squared (η_p_^2^). For simplicity of reporting, age groups are referred to according to the mean age of the group (for example, 12y refers to 12-year-olds, the Year 7 participants whose ages ranged between 11.75 and 12.67). Main effects of Age group were followed up with three planned tests assessing differences between 12y and 15y, 13y and 15y, and 14y and 15y, since the greatest differences were anticipated in comparison to the oldest group.

In the science and maths misconceptions task, three participants were excluded due to low accuracy (one 13y) or slow RT (one 12y, one 13y), leaving a final *N* = 87 participants. Two (Trial type: control, misconception) x two (Discipline: science, maths) x two (Statement type: true, false) x four (Age group: 12y, 13y, 14y, 15y) mixed model repeated measures ANOVAs were performed on accuracy and RT. Three participants were excluded from the simple Go/No-Go task, and two participants were excluded from the complex Go/No-Go tasks (see S1 File). Analyses of age effects in the three tasks are reported in S1 File.

Participants excluded from any individual task analysis were also excluded from the regression analyses (final *n*s: 12y: *n* = 20, 13y: *n* = 22, 14y: *n* = 21, 15y: *n* = 18), leaving a total *N* = 81. Correlations were run between the variables of interest to examine collinearity and assess associations between measures across the whole sample. Hierarchical multiple regressions investigated whether inhibitory control variables could account for individual differences in science and maths misconception accuracy and RT.

Regression models added the control variables using the enter function in block 1: age in months, WASI Vocabulary and Matrix Reasoning raw scores, and science and maths control performance. These variables were expected to have an influence on the outcome measure, but were not the primary predictive variables of interest. Raw WASI scores were entered rather than standardised scores so that scores were directly comparable across ages. Go/No-Go variables were entered stepwise in block 2: simple No-Go accuracy, complex No-Go accuracy, simple Go accuracy, complex Go accuracy, simple Go RT, complex Go RT. Stroop variables were entered stepwise in block 3: accuracy cost (congruent minus incongruent), RT cost (incongruent minus congruent), congruent accuracy, and congruent RT.

Inclusion of separate Go/No-Go and Stroop blocks allowed for investigation of variance explained individually by response and semantic inhibition. Stepwise entry and the inclusion of variables that do not necessarily reflect inhibition (such as Go accuracy or congruent Stroop RT) enabled examination of the possibility that general processing speed or accuracy alone were the most important predictors of performance, rather than inhibition per se.

Follow up exploratory regressions were run on science and maths separately, to examine possible discipline-specific effects and to explore whether directions of association were consistent. All follow up models included the control variables and the inhibitory control variables identified in the science and maths combined regressions, using the enter method.

## Results

### Science and maths misconceptions

In line with the design of this task, participants tended to give the correct answer in control trials, with a mean accuracy of 82.2% (Table 2), while they made more errors on misconception trials, where the mean accuracy was 54.7%. While this is close to chance performance (50%), S2 Fig shows a histogram of mean accuracy in each of the 96 science and maths misconception trials demonstrating that participants answered correctly more often on some trials than others. This indicates that the accuracy in misconception trials is not attributable to chance performance (guesses) on all problems.

**Table 2:** Accuracy and RT estimated marginal means in the science and maths misconceptions task.

		Accuracy (%)	RT (ms)
		*M (SE)*	*M (SE)*
**Main effects**			
*Trial type*		*F*(1, 83) = 816.73, *p* < .001, η_p_^2^ = .908	*F*(1, 83) = 310.32, *p* < .001, η_p_^2^ = .789
Control		82.2 (0.8)	5156 (134)
Misconception		54.7 (0.9)	6683 (190)
*Discipline*		n.s., *p* = .367	*F*(1, 83) = 55.73, *p* < .001, η_p_^2^ = .402
Science		68.1 (0.9)	5598 (147)
Maths		68.9 (0.7)	6240 (180)
*Statement type*		*F*(1, 83) = 38.64, *p* < .001, η_p_^2^ = .318	*F*(1, 83) = 5.26, *p* = .024, η_p_^2^ = .060
True		72.5 (0.8)	5837 (158)
False		64.4 (1.1)	6002 (168)
*Age group*		*F*(3, 83) = 5.61, *p* = .001, *η*_p_^2^ = .169	n.s., *p* = .631
12y		65.3 (1.3)	6149 (301)
13y		66.5 (1.3)	5856 (307)
14y		69.2 (1.4)	6073 (322)
15y		72.8 (1.5)	5601 (338)
**Interaction effects**			
*Trial type*	*Statement type*	*F*(1, 83) = 11.48, *p* = .001, η_p_^2^ = .121	n.s., *p* = .076
Control	True	84.8 (0.9)	4986 (136)
	False	79.6 (1.1)	5327 (153)
Misconception	True	60.2 (1.1)	6688 (199)
	False	49.3 (1.4)	6678 (204)
*Discipline*	*Statement type*	*F*(1, 83) = 67.73, *p* < .001, η_p_^2^ = .449	*F*(1, 83) = 14.15, *p* < .001, η_p_^2^ = .146
Science	True	75.8 (1.1)	5406 (146)
	False	60.3 (1.4)	5791 (159)
Maths	True	69.3 (0.9)	6268 (181)
	False	68.5 (1.1)	6214 (193)

A two (Trial type: control, misconception) x two (Discipline: science, maths) x two (Statement type: true, false) x four (Age group: 12y, 13y, 14y, 15y) mixed model repeated measures ANOVA performed on accuracy showed main effects of Trial type and Statement type, with greater accuracy in control compared to misconception trials, and true compared to false statements (Table 2). There was no main effect of Discipline.

These main effects were modulated by a significant interaction between Trial type and Statement type (Table 2), which was followed up with two repeated measures ANOVAs on control and misconception accuracy. The interaction was attributable to less difference in accuracy between true and false statements in control trials, *F*(1,83) = 11.33, *p* = .001, η_p_^2^ = .120, compared to misconception trials, *F*(1,83) = 28.57, *p* < .001, η_p_^2^ = .256. There was an additional significant interaction between Discipline and Statement type, whereby the difference between true and false statements was significant for science trials, *F*(1,83) = 84.75, *p* < .001, η_p_^2^ = .505, but not maths trials, *p* = .629 (Table 2). Accuracy increased with age and the pattern of age increase differed between science and maths (see S1 File for a description of Age group effects on accuracy).

The same ANOVA was performed on RT. There were main effects of Trial type, Discipline, and Statement type, with longer RTs in maths compared to science, misconception compared to control trials, and false compared to true trials (Table 2). There was no main effect of Age group on RT. As for accuracy, there was a significant interaction between Discipline and Statement type, which was followed up with two further repeated measures ANOVAs. There was a significant difference between true and false statements for science, *F*(1,83) = 20.50, *p* < .001, η_p_^2^ = .198, with longer RTs for false trials (Table 2), while there was no significant difference between true and false trials for maths, *p* = .589. No other interaction was significant, *p’*s > .1.

In summary, the main finding of interest was lower accuracy and longer RTs in misconception compared to control trials. This is consistent with the hypothesis and design of the paradigm, since it was anticipated that intuitive responses would be incorrect and reasoning would take longer in misconception trials. Lower accuracy and longer RTs were also found for false statements compared to true statements in science, but not maths. Maths RTs were longer than science RTs overall. Finally, improved performance with age was reflected in accuracy only and more prolonged in science than maths.

### Inhibitory control

The results of the inhibitory control tasks are summarised in Table 3 and detailed in S1 File. Briefly, participants showed the expected poorer performance in No-Go trials of the Go/No-Go tasks and in incongruent trials of the numerical Stroop, reflecting the inhibitory control demands of these trials.

**Table 3:** Accuracy and RT estimated marginal means in the inhibitory control tasks.

	Simple Go/No-Go	Complex Go/No-Go	Numerical Stroop
	*M (SE)*	*M (SE)*	*M (SE)*
**Accuracy (%)**			
*Trial type*			
Go/Congruent	96.4 (0.4)	84.5 (1.0)	96.1 (0.4)
No-Go/Incongruent	84.7 (1.3)	53.6 (2.0)	80.1 (1.2)
*Age group*			
12y	86.7 (1.4)	69.2 (2.2)	84.3 (1.3)
13y	91.5 (1.4)	67.7 (2.1)	89.2 (1.3)
14y	92.4 (1.5)	68.2 (2.3)	89.5 (1.4)
15y	91.4 (1.6)	71.3 (2.5)	89.4 (1.5)
**RT (ms)**			
*Trial type*			
Go/Congruent	346 (3)	400 (6)	671 (9)
No-Go/Incongruent	^a^	^a^	779 (11)
*Age group*			
12y	354 (7)	400 (11)	745 (18)
13y	353 (7)	399 (10)	752 (19)
14y	348 (7)	413 (11)	700 (20)
15y	330 (7)	388 (12)	703 (21)

^a^ RTs are for correct trials only, therefore there are no RTs for No-Go trials.

### Regression analyses

Correlations between the variables of interest were examined (Table B in S1 File) and assumptions regarding multicollinearity were met. An initial hierarchical multiple regression (Table 4) investigated whether inhibitory control measures could account for variance in science and maths misconception accuracy. The first model (1a) with age, WASI Vocabulary and Matrix Reasoning raw scores, and science and maths control accuracy as predictors, was significant, explaining 26% of the variance. Age and science and maths control accuracy were significant predictors of misconception accuracy. Stroop RT cost was selected using a stepwise approach in model 1b, uniquely accounting for 5% of the variance. Greater Stroop RT cost was associated with lower misconception accuracy. No Go/No-Go variables were selected by the model.

**Table 4:** Regression models for science and maths combined.

	Variables	β	*t*	*p*
DV: Science and maths misconception accuracy				
Model 1a	Constant		-0.62	.535
*F*(4, 76) = 6.61, *p* < .001, *R*^2^ = 26%	**Age (months)**	**.29**	**2.83**	**.006**
	WASI Vocabulary raw	.10	0.85	.397
	WASI Matrix Reasoning raw	.09	0.87	.389
	**Science and maths control accuracy**	**.26**	**2.22**	**.029**
Model 1b	Constant		-0.24	.812
*F*(5, 75) = 6.68, *p* < .001, *R*^2^ = 31%, *ΔR*^*2*^ = 5.0%	**Age (months)**	**.30**	**3.02**	**.004**
	WASI Vocabulary raw	.08	0.71	.482
	WASI Matrix Reasoning raw	.09	0.87	.389
	**Science and maths control accuracy**	**.26**	**2.28**	**.026**
	**Stroop RT cost**	**-.22**	**-2.33**	**.023**
DV: Science and maths misconception RT				
Model 2a	Constant		-1.37	.174
*F*(4, 76) = 101.52, *p* < .001, *R*^2^ = 84%	Age (months)	-.01	-0.23	.819
	WASI Vocabulary raw	.09	1.89	.063
	WASI Matrix Reasoning raw	.07	1.46	.148
	**Science and maths control RT**	**.911**	**19.61**	**< .001**
Model 2b	Constant		-1.73	.088
*F*(5, 75) = 86.61, *p* < .001, *R*^2^ = 85%, *ΔR*^*2*^ = 1.0%	Age (months)	-.01	-0.12	.908
	WASI Vocabulary raw	.08	1.66	.101
	WASI Matrix Reasoning raw	.07	1.48	.143
	**Science and maths control RT**	**.91**	**20.05**	**< .001**
	**Complex No-Go accuracy**	**.10**	**2.26**	**.027**

Significant predictors (*p* < .05) are highlighted in bold. DV = dependent variable; β = standardised coefficients.

The second regression investigated misconception RT. The first model (2a) with age, WASI Vocabulary and Matrix Reasoning raw scores, and science and maths control RT as predictors, was significant, explaining 84% of the variance. Only science and maths control RT was a significant predictor of misconception accuracy. Complex No-Go accuracy was selected in model 2b, uniquely accounting for 1% of the variance. Greater complex No-Go accuracy was associated with higher misconception RT. No Stroop variables were selected by the model.

Follow up exploratory regressions (Table 5) examined the extent to which these associations held for science and maths individually, adding the inhibitory control variables with the enter method. Stroop RT cost was not a significant predictor of science (model 3) or maths (model 4) misconception accuracy, although the *p*-values were at trend and the coefficients were in the same direction as the combined analyses. Complex Go/No-Go accuracy was not a significant predictor of science (model 5) or maths (model 6) misconception RT. This time the coefficient was positive for maths, as with the combined analyses, but negative for science.

**Table 5:** Regression models for science and maths separately.

	Variables	β	*t*	*p*
DV: Science misconception accuracy				
Model 3	Constant		-0.21	.904
*F*(5, 75) = 4.21, *p* = .002, *R*^2^ = 22%	**Age (months)**	**.21**	**2.00**	**.049**
	WASI Vocabulary raw	-.02	-0.19	.849
	WASI Matrix Reasoning raw	.00	.00	.999
	**Science control accuracy**	**.35**	**2.98**	**.004**
	Stroop RT cost	-.18	-1.77	.080
DV: Maths misconception accuracy				
Model 4	Constant		.049	.961
*F*(5, 75) = 3.98, *p* < .003, *R*^2^ = 21%	**Age (months)**	**.27**	**2.52**	**.014**
	WASI Vocabulary raw	.15	1.30	.199
	WASI Matrix Reasoning raw	.15	1.42	.160
	Maths control accuracy	.09	.76	.452
	Stroop RT cost	-.18	-1.76	.083
DV: Science misconception RT				
Model 5	Constant		-0.11	.915
*F*(5, 75) = 32.84, *p* < .001, *R*^2^ = 69%	Age (months)	.08	1.28	.203
	WASI Vocabulary raw	.03	0.37	.711
	WASI Matrix Reasoning raw	.01	0.14	.890
	**Science control RT**	**.84**	**12.43**	**< .001**
	Complex No-Go accuracy	-.10	-1.55	.124
DV: Maths misconception RT				
Model 6	Constant		-.79	.43
*F*(5, 75) = 43.02, *p* < .001, *R*^2^ = 74%	Age (months)	-.08	-1.31	.195
	WASI Vocabulary raw	.11	1.78	.079
	WASI Matrix Reasoning raw	.11	1.86	.067
	**Maths control RT**	**.83**	**13.96**	**< .001**
	Complex No-Go accuracy	.063	1.05	.296

Significant predictors (*p* < .05) are highlighted in bold. DV = dependent variable; β = standardised coefficients.

In summary, the regression analyses revealed unique roles for response and semantic inhibition in reasoning about science and maths misconceptions. Both response inhibition (complex No-Go accuracy) and semantic inhibition (Stroop RT cost) were predictors of performance when science and maths misconceptions were combined. Proficiency in semantic inhibition was more important for predicting misconception accuracy, while proficient response inhibition was more important for predicting longer RTs when addressing misconception problems.

## Discussion

The current study investigated the role of inhibitory control in counterintuitive science and maths reasoning in adolescence. It was hypothesised that better inhibitory control would be associated with better performance in science and maths misconception problems, when controlling for performance on related problems, age, and general cognitive ability. Ninety adolescents were tested on response and semantic inhibition and a novel science and maths misconceptions task. Both response and semantic inhibition were associated with performance in science and maths misconception trials, beyond performance in control trials and individual differences in general cognitive ability or age. This was the first study to consider the unique roles of response and semantic inhibition in this context, demonstrating that response inhibition may be more related to RTs in counterintuitive reasoning, while semantic inhibition may be more related to accuracy. General performance on the science and maths misconceptions task will be considered first, followed by inhibitory control findings; finally the association between inhibitory control and science and maths misconception performance will be discussed.

### Science and maths misconceptions

As anticipated, accuracy was lower and RTs slower for misconception trials, indicating that reasoning about counterintuitive curriculum-related concepts leads to misconceptions in this age group, even in the oldest participants who should have a good understanding of these concepts having covered them all at school. Only small age effects were observed, in line with standardised assessment findings that only small improvements are made in maths within this age range [6].

The reduction in accuracy in false trials compared to true trials was greater for misconception than control trials, which may be due to increased cognitive demand in false trials. To arrive at the correct response, the participant must first read the statement and detect an error, then possibly generate the true statement internally before deciding that the statement presented is false. This may explain why it is easier to answer a true statement correctly, especially if it is counterintuitive. This pattern of performance was observed in science trials only, which may be explained by the inclusion in maths of nine problem-sets containing equations, where both true and false trials require a mental calculation, which should limit any specific increase in cognitive demand for false trials. It should also be noted that a higher proportion of science trials were accompanied by a picture (79% vs. 50%) which cannot be ruled out as a source of difference between the two disciplines.

Overall, these findings support the previous literature that misconceptions due to intuitive reasoning exist in this age range [7,54]. Although we used a novel task, which has not been extensively validated, the inclusion of problems that cover the curriculum broadly is a strength of the study, allowing greater generalisation and relevance for education.

### Inhibitory control

The inhibitory control tasks showed a degree of improvement with age, echoing findings in the literature [22]. Some measures of inhibitory control were moderately correlated with each other, with the highest correlations between RT measures, likely representing processing speed [57] rather than inhibition per se. There was a marginal negative correlation between the two inhibitory control measures that were selected by the regression model: Higher complex response inhibition accuracy was associated with lower semantic inhibition RT cost. This suggests that response and semantic inhibition are partially related, in keeping with previous literature [26], whereby the ability to make less impulsive motor responses is linked to the ability to suppress irrelevant stimuli with less interference.

### The role of inhibitory control in counterintuitive science and maths reasoning

Both response and semantic inhibition were associated with science and maths misconception performance when controlling for age, general cognitive ability, and control performance. In line with our first hypothesis, a smaller difference in RT between incongruent and congruent Stroop trials, suggesting less interference and better semantic inhibition, was associated with higher accuracy on misconception trials. These results fit with the proposal that semantic inhibition may allow suppression of naïve beliefs or irrelevant perceptual information in order to reach the correct answer to counterintuitive problems. Note that while the Stroop task was selected to measure semantic inhibition, it requires suppression of a motor response to some extent [58], so this measure may partly implicate response inhibition. Although the amount of variance explained was small, it is still meaningful given that the model included age, general cognitive ability (verbal and non-verbal), and performance in related science and maths control trials. The fact that the association is observed after inclusion of control trials as a covariate in the analyses is consistent with the idea that semantic inhibitory control may play a specific (or more important) role in science and maths counterintuitive reasoning rather than science and maths reasoning more broadly.

The ability to withhold a response in the complex Go/No-Go was associated with longer RTs on misconception trials. Therefore, contrary to our hypothesis, good response inhibition is not associated with better performance in the science and maths misconceptions task. However, a possible interpretation is that good response inhibition may afford more time for consideration of the response, with a less impulsive pattern of responding. Individuals may not necessarily eventually choose the correct response, but they may be able to spend more time thinking about their response and evaluate competing alternative answers.

The complex Go/No-Go measured inhibitory control within the context of a cognitive load. The regression model’s selection of a complex rather than simple Go/No-Go variable implicates individual differences in the ability to manage combined response inhibition and working memory demands. The use of this ability is exemplified by a maths misconception problem that requires counting items and calculating probabilities, and holding this information in mind while considering how it applies to the statement. This account is consistent with suggestions that beliefs must be held in working memory during reasoning, before the incorrect response is inhibited [19]. Future work could measure a purer form of working memory in a separate task to assess the extent to which working memory makes a unique contribution outside of the context of inhibitory control.

The discipline-specific analyses had reduced power due to the smaller number of trials, in addition to the different number of pictures within the stimuli, so must be interpreted cautiously. There were also fewer maths problems than science problems. The results overall suggest that misconceptions in science and maths show similar associations to semantic inhibition, with potentially different associations with response inhibition: Higher complex No-Go accuracy was associated with shorter responses in science but longer response in maths. However, these exploratory analyses did not reach significance so further research would be necessary to determine discipline-specific associations. Further distinctions could also be made within disciplines. For example, although not focusing on counterintuitive reasoning, previous research found an association between numerical dot comparison inhibition and procedural maths but not conceptual or factual maths in adolescents [20].

It is possible that the completion of the inhibitory control tasks before the science and maths task may have led to increased inhibitory control use in the latter due to practice effects. Nonetheless, short breaks between tasks, which included talking to the researcher, may have dissipated any such effects. Further, any effect will have applied to all participants, and there remains poorer accuracy in misconception problems compared to control problems.

Finally, future work should consider the possibility that different types of inhibition specifically allow the suppression of different types of misconception. The current study did not categorise types of misconception, and contained a mixture of those due to misleading perceptual cues, previously held beliefs, and prior experiences. While the focus here was on covering a broad set of problems, it would be interesting to establish specific links between types of inhibitory control and misconceptions of different origins. Further, intervention studies could assess the impact of inhibitory control training in the context of science and maths learning. This would help to establish the extent to which the association between inhibitory control and counterintuitive reasoning is a causal one; something that the current paper could not address directly. The evidence so far suggests that encouraging learners to inhibit their immediate responses might lead to improved counterintuitive reasoning in science and maths.

## Conclusions

The associations seen in this novel study suggest that both response inhibition and semantic inhibition play a role in counterintuitive science and maths reasoning problems that are curriculum-related. The results are in line with the idea that inhibition is required to overcome misleading perceptual cues [45] or old theories that are still present [13,17], and that old theories remain present [1,14], rather than being replaced or restructured through learning [3,8]. As argued by Houdé [15], this study provides further evidence that poor reasoning partly reflects poor inhibitory control as opposed to simply poor logic or understanding. The present study focused on curriculum-related science and maths problems and adolescence. The results are therefore relevant for secondary education and suggest that individual differences in inhibitory control may play a role in science and maths academic outcome.

## Supporting information

S1 Fig Example problem-sets in (a) science and (b) maths. (TIF)

S2 Fig Histogram of mean accuracy in the 96 science and maths misconception problems. The range of mean accuracy observed suggests that although the mean overall *Misconception* trials accuracy was near chance at 54.7%, participants did not consistently guess across all problems.
(TIF)

S1 File Inhibitory control and counterintuitive science and maths reasoning in adolescence: Supporting information. This file contains the method and results of the inhibitory control tasks, and further accuracy analyses from the science and maths misconceptions task.
(DOCX)

## References

[1] Mareschal D. The neuroscience of conceptual learning in science and mathematics. Curr Opin Behav Sci. 2016;10: 114–118. doi: 10.1016/j.cobeha.2016.06.001

[2] Carey S. Science education as conceptual change. J Appl Dev Psychol. 2000;21(1): 13–19. doi: 10.1016/S0193-3973(99)00046-5

[3] Vosniadou S. Conceptual change and education. Hum Dev. 2007;50(1): 47–54. doi: 10.1159/000097684

[4] McNeil NM, Alibali MW. Why won’t you change your mind? Knowledge of operational patterns hinders learning and performance on equations. Child Dev. 2005; 76(4): 883–899. doi: 10.1111/j.1467-8624.2005.00884.x 16026503 10.1111/j.1467-8624.2005.00884.x

[5] Rousselle L, Palmers E, Noël M-P. Magnitude comparison in preschoolers: What counts? Influence of perceptual variables. J Exp Child Psychol. 2004;87(1): 57–84. doi: 10.1016/j.jecp.2003.10.005 14698689 10.1016/j.jecp.2003.10.005

[6] Ryan J, Williams J. Children’s mathematics 4–15: Learning from errors and misconceptions. Maidenhead, UK: Open University Press; 2007.

[7] Stavy R, Tirosh D. How students (mis-)understand science and mathematics: Intuitive Rules. New York: Teachers College Press; 2000.

[8] Posner GJ, Strike KA, Hewson PW, Gertzog WA. Accommodation of a scientific conception: Toward a theory of conceptual change. Sci Educ. 1982;66(2): 211–227. doi: 10.1002/sce.3730660207

[9] Allen M. Misconceptions in primary science. 2nd ed Maidenhead, UK: Open University Press; 2014.

[10] Vosniadou S. Examining cognitive development from a conceptual change point of view: The framework theory approach. Eur J Dev Psychol. 2014;11(6): 645–661. doi: 10.1080/17405629.2014.921153

[11] Shtulman A, Valcarcel J. (2012). Scientific knowledge suppresses but does not supplant earlier intuitions. Cognition, 2012;124: 209–215. doi: 10.1016/j.cognition.2012.04.005 22595144 10.1016/j.cognition.2012.04.005

[12] Borst G, Poirel N, Pineau A, Cassotti M, Houdé O. Inhibitory control efficiency in a Piaget-like class-inclusion task in school-age children and adults: A developmental negative priming study. Dev Psychol. 2013;49(7): 1366–1374. doi: 10.1037/a0029622 22889392 10.1037/a0029622

[13] Brault Foisy L-M, Potvin P, Riopel M. Masson S. Is inhibition involved in overcoming a common physics misconception in mechanics? Trends Neurosci Educ. 2015; 4(1): 26–36. doi: 10.1016/j.tine.2015.03.001

[14] Dunbar KN, Fugelsang JA, Stein C. Do naïve theories ever go away? Using brain and behaviour to understand changes in concepts In: Lovett M, Shah P, editors. Thinking with data. New Jersey, USA: Lawrence Erlbaum Associates; 2007 pp. 193–205.

[15] Houdé O. Inhibition and cognitive development: Object, number, categorization, and reasoning. Cogn Dev. 2000;15: 63–73. doi: 10.1016/S0885-2014(00)00015-0

[16] Lubin A, Vidal J, Lanoë C, Houdé O, Borst G. Inhibitory control is needed for the resolution of arithmetic word problems: A developmental negative priming study. J Educ Psychol. 2013;105(3): 701–708. doi: 10.1037/a0032625

[17] Masson S, Potvin P, Riopel M, Brault Foisy L-M. (2014). Differences in brain activation between novices and experts in science during a task involving a common misconception in electricity. Mind Brain Educ. 2014;8(1): 44–55. doi: 10.1111/mbe.12043

[18] Lortie-Forgues H, Tian J, Siegler RS. (2015). Why is learning fraction and decimal arithmetic so difficult? Dev Rev. 2015;38: 201–221. doi: 10.1016/j.dr.2015.07.008

[19] Zaitchik D, Iqbal Y, Carey S. The effect of executive function on biological reasoning in young children: An individual differences study. Child Dev. 2014;85(1): 160–175. doi: 10.1111/cdev.12145 23889035 10.1111/cdev.12145

[20] Gilmore C, Keeble S, Richardson S, Cragg L. The role of cognitive inhibition in different components of arithmetic. ZDM. 2015;47: 771–782. doi: 10.1007/s11858-014-0659-y

[21] Merkley R, Thompson J, Scerif G. (2015). Of huge mice and tiny elephants: Exploring the relationship between inhibitory processes and preschool maths skills. Front Psychol. 2015;1903(6): 1–14. doi: 10.3389/fpsyg.2015.01903 26779057 10.3389/fpsyg.2015.01903 PMC4703825

[22] Leon-Carrion J, García-Orza J. Pérez-Santamaría FJ. Development of the inhibitory component of the executive functions in children and adolescents. Int J Neurosci. 2004;114(10): 1291–1311. doi: 10.1080/00207450490476066 15370187 10.1080/00207450490476066

[23] Department for Education. (2013a). Mathematics programmes of study: Key stage 3.

[24] Department for Education. (2013b). Science programmes of study: Key stage 3.

[25] Nigg JT. On inhibition/disinhibition in developmental psychopathology: Views from cognitive and personality psychology and a working inhibition taxonomy. Psychol Bull. 2000;126(2): 220–246. 10748641 10.1037/0033-2909.126.2.220

[26] Verbruggen F, Liefooghe B, Vandierendonck A. The interaction between stop signal inhibition and distractor interference in the flanker and Stroop task. Acta Psychol. 2004;116(1): 21–37. doi: 10.1016/j.actpsy.2003.12.011 15111228 10.1016/j.actpsy.2003.12.011

[27] Simmonds DJ, Pekar JJ, Mostofsky SH. Meta-analysis of go/no-go tasks demonstrating that fMRI activation associated with response inhibition is task-dependent. Neuropsychologia. 2008;46(1): 224–232. doi: 10.1016/j.neuropsychologia.2007.07.015 17850833 10.1016/j.neuropsychologia.2007.07.015 PMC2327217

[28] Stroop JR. Studies of interference in serial verbal reactions. J Exp Psychol. 1935;18(6): 643–662. doi: 10.1037/h0054651

[29] Humphrey G, Dumontheil I. Development of risk-taking, perspective-taking, and inhibitory control during adolescence. Dev Neuropsychol. 2016;5641: 1–18. doi: 10.1080/87565641.2016.1161764 27070826 10.1080/87565641.2016.1161764

[30] Tamm L, Menon V, Reiss AL. Maturation of brain function associated with response inhibition. J Am Acad Child Adolesc Psychiatry. 2002;41(10): 1231–1238. doi: 10.1097/00004583-200210000-00013 12364845 10.1097/00004583-200210000-00013

[31] Comalli PE Jr, Wapner S, Werner H. Interference effects of Stroop color-word test in childhood, adulthood, and aging. J Genet Psychol. 1962;100: 47–53. doi: 10.1080/00221325.1962.10533572 13880724 10.1080/00221325.1962.10533572

[32] Miyake A, Friedman NP, Emerson MJ, Witzki AH, Howerter A, Wager TD. The unity and diversity of executive functions and their contributions to complex “frontal lobe” tasks: A latent variable analysis. Cogn Psychol. 2000;41(1): 49–100. doi: 10.1006/cogp.1999.0734 10945922 10.1006/cogp.1999.0734

[33] Huizinga M, Dolan CV, van der Molen MW. Age-related change in executive function: Developmental trends and a latent variable analysis. Neuropsychologia. 2006; 44(11): 2017–2036. doi: 10.1016/j.neuropsychologia.2006.01.010 16527316 10.1016/j.neuropsychologia.2006.01.010

[34] Khng KH, Lee K. The relationship between Stroop and stop-signal measures of inhibition in adolescents: Influences from variations in context and measure estimation. PLoS One. 2014;9(7): e101356 doi: 10.1371/journal.pone.0101356 24992683 10.1371/journal.pone.0101356 PMC4081588

[35] Baker ST, Gjersoe NL, Sibielska-Woch K, Leslie AM, Hood BM. Inhibitory control interacts with core knowledge in toddlers’ manual search for an occluded object. Dev Sci. 2011;14: 270–279. doi: 10.1111/j.1467-7687.2010.00972.x 22213900 10.1111/j.1467-7687.2010.00972.x

[36] Hood B, Cole-Davies V, Dias M. Looking and search measures of object knowledge in preschool children. Dev Psychol. 2003;39(1): 61–70. doi: 10.1037/0012-1649.39.1.61 12518809

[37] Mayer D, Sodian B, Koerber S, Schwippert K. Scientific reasoning in elementary school children: Assessment and relations with cognitive abilities. Learn Instr. 2014;29: 43–55. doi: 10.1016/j.learninstruc.2013.07.005

[38] Rhodes SM, Booth JN, Campbell LE, Blythe RA, Wheate NJ, Delibegovic M. Evidence for a role of executive functions in learning biology: Executive functions and science. Infant Child Dev. 2014;23: 67–83. doi: 10.1002/icd.1823

[39] Rhodes SM, Booth JN, Palmer LE, Blythe RA, Delibegovic M, Wheate NJ. (2016). Executive functions predict conceptual learning of science. Br J Dev Psychol, 34, 261–275. doi: 10.1111/bjdp.12129 26751597 10.1111/bjdp.12129

[40] Siegler RS. Children’s Thinking. 3rd ed Upper Saddle River, NJ: Prentice Hall; 1998.

[41] Siegler RS, Jenkins E. How children discover new strategies. New Jersey: Lawrence Erlbaum Associates, Inc; 1989.

[42] Khng KH, Lee K. Inhibiting interference from prior knowledge: Arithmetic intrusions in algebra word problem solving. Learn Individ Differ. 2009;19(2): 262–268. doi: 10.1016/j.lindif.2009.01.004

[43] Monette S, Bigras M, Guay M-C. The role of executive functions in school achievement at the end of Grade 1. J Exp Child Psychol. 2011;109(2): 158–173. doi: 10.1016/j.jecp.2011.01.008 21349537 10.1016/j.jecp.2011.01.008

[44] Allan NP, Hume LE, Allan DM, Farrington AL, Lonigan CJ. Relations between inhibitory control and the development of academic skills in preschool and kindergarted: A meta-analysis. Dev Psychol. 2014:50(10): 2368–2379. doi: 10.1037/a0037493 25069051 10.1037/a0037493

[45] Linzarini A, Houdé O, Borst G. When Stroop helps Piaget: An inter-task positive priming paradigm in 9-year-old children. J Exp Child Psychol. 2015;139: 71–82. doi: 10.1016/j.jecp.2015.05.010 26086072 10.1016/j.jecp.2015.05.010

[46] Fugelsang JA, Dunbar KN. Brain-based mechanisms underlying complex causal thinking. Neuropsychologia. 2005;45(8): 1204–1203. doi: 10.1016/j.neuropsychologia.2004.10.012 15817178 10.1016/j.neuropsychologia.2004.10.012

[47] Goel V, Dolan RJ. Explaining modulation of reasoning by belief. Cognition. 2003;87(1): B11–B22. doi: 10.1016/S0010-0277(02)00185-3 12499108 10.1016/s0010-0277(02)00185-3

[48] Prado J, Noveck IA. Overcoming perceptual features in logical reasoning: a parametric functional magnetic resonance imaging study. J Cogn Neurosci. 2007;19(4): 642–657. doi: 10.1162/jocn.2007.19.4.642 17381255 10.1162/jocn.2007.19.4.642

[49] Stavy R, Babai R. Overcoming intuitive interference in mathematics: insights from behavioral, brain imaging and intervention studies. ZDM. 2010;42(6): 621–633. doi: 10.1007/s11858-010-0251-z 10.1007/s10339-018-0893-2 30443818

[50] Houdé O, Zago L, Mellet E, Moutier S, Pineau A, Mazoyer B, et al Shifting from the perceptual brain to the logical brain: The neural impact of cognitive inhibition training. J Cogn Neurosci. 2000;12(5); 721–728. doi: 10.1162/089892900562525 11054915 10.1162/089892900562525

[51] Parsons R, Gannon P. KS3 science: Complete study and practice. Newcastle upon Tyne, UK: Coordination Group Publications Ltd; 2014.

[52] Parsons R. KS3 maths: Complete study and practice. Newcastle upon Tyne, UK: Coordination Group Publications Ltd; 2014.

[53] Watanabe J, Sugiura M, Sato K, Sato Y, Maeda Y, Matsue Y, et al The human prefrontal and parietal association cortices are involved in No-Go performances: An event-related fMRI study. Neuroimage. 2002;17(3): 1207–1216. doi: 10.1006/nimg.2002.1198 12414261 10.1006/nimg.2002.1198

[54] Driver R, Squires A, Rushworth P, Wood-Robinson V. Making sense of secondary science: Research into children’s ideas. Classic ed New York: Routledge; 2015.

[55] Wechsler D. Wechsler abbreviated scale of intelligence (WASI-II). 2nd ed San Antonio, TX: Pearson; 2011.

[56] Field A. Linear models: Looking for bias. 2012. http://www.statisticshell.com/docs/linearmodelsbias.pdf

[57] Kail R. Processing time decreases globally at an exponential rate during childhood and adolescence. J Exp Child Psychol. 1993;56(2): 254–265. doi: 10.1006/jecp.1993.1034 8245769 10.1006/jecp.1993.1034

[58] van Veen V, Carter CS. Separating semantic conflict and response conflict in the Stroop task: A functional MRI study. Neuroimage. 2005;27(3): 495–504. doi: 10.1016/j.neuroimage.2005.04.042 15964208 10.1016/j.neuroimage.2005.04.042

