# Supplementary figures and images for: Inhibitory control and counterintuitive science and maths reasoning in adolescence
Source: PLoS One. 2018 Jun 21;13(6):e0198973. doi: 10.1371/journal.pone.0198973 (PMC6013119; doi:10.1371/journal.pone.0198973)

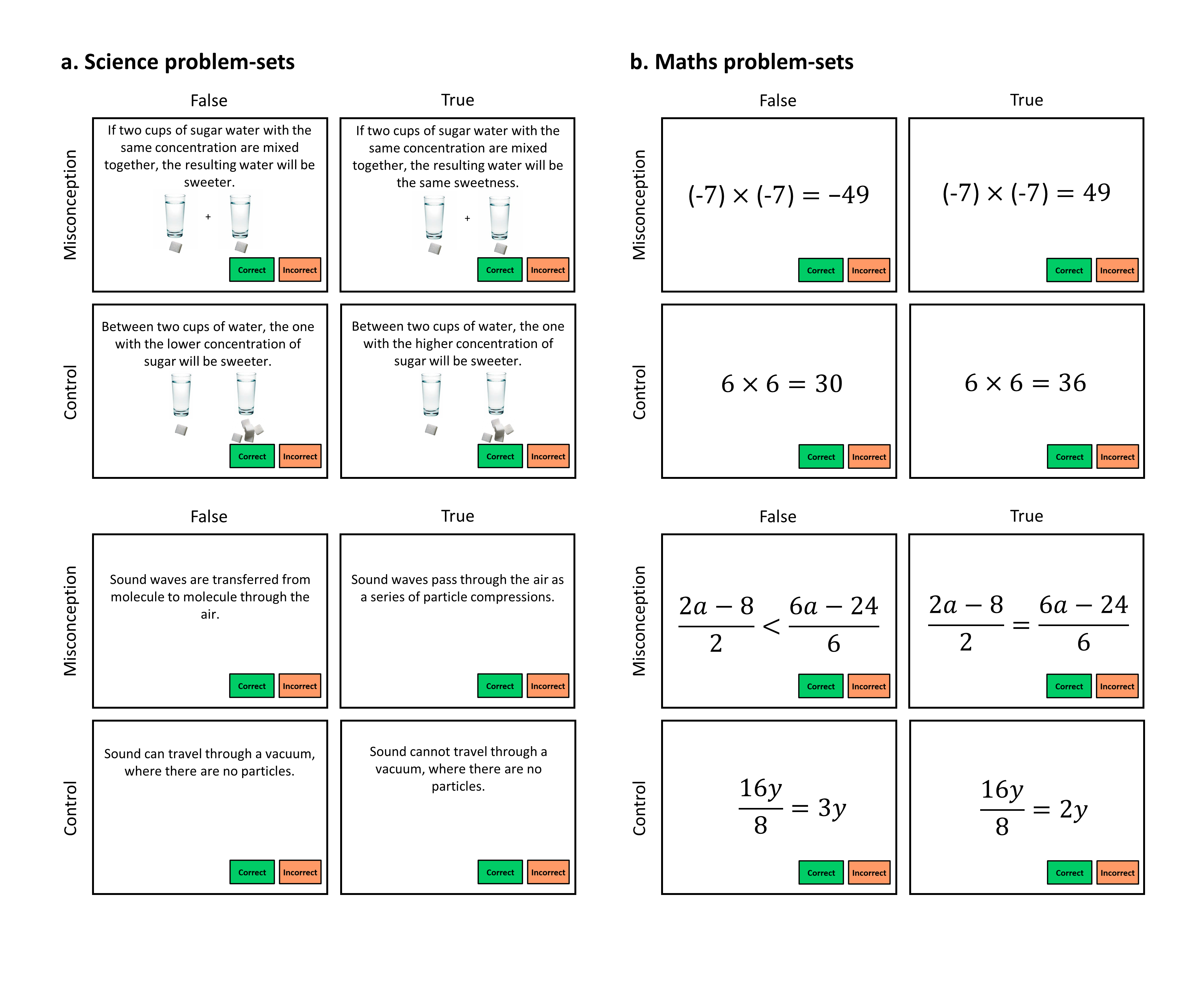

Supplement: S1 Fig — (TIF) [file pone.0198973.s001.tif]

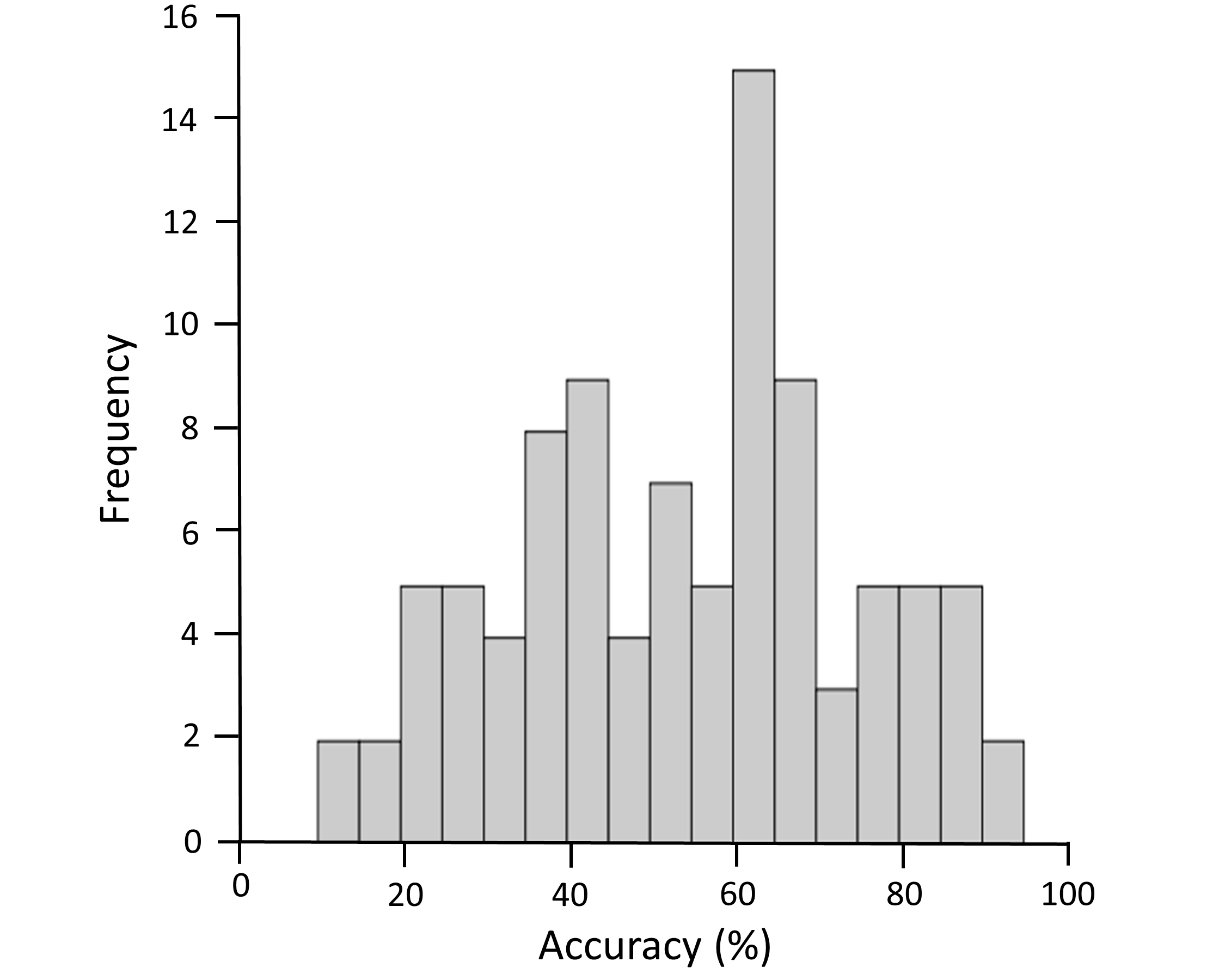

Supplement: S2 Fig — The range of mean accuracy observed suggests that although the mean overall Misconception trials accuracy was near chance at 54.7%, participants did not consistently guess across all problems. (TIF) [file pone.0198973.s002.tif]
