# Supplementary material for: Inhibitory control and counterintuitive science and maths reasoning in adolescence
Source: PLoS One. 2018 Jun 21;13(6):e0198973. doi: 10.1371/journal.pone.0198973 (PMC6013119; doi:10.1371/journal.pone.0198973)
Supplement: S1 File — This file contains the method and results of the inhibitory control tasks, and further accuracy analyses from the science and maths misconceptions task. (DOCX) [file pone.0198973.s003.docx]

**Inhibitory control and counterintuitive science and maths reasoning in adolescence: Supporting information (S1)**

Here we present the method and results of the inhibitory control tasks, and further accuracy analyses from the science and maths misconceptions task, from the paper ‘*Inhibitory control and counterintuitive science and maths reasoning in adolescence*’.

**Supplementary methods**

**Inhibitory control tasks**

The simple and complex Go/No-Go tasks were computerised measures of response inhibition adapted from Watanabe et al. [1]. Familiarisation and practice phases for both tasks consisted of ten trials. In the familiarization phase, coloured squares (green squares in the simple task; yellow or blue squares in the complex task) appeared on the left or right of the screen, and participants pressed the corresponding key, using their index or middle finger, to indicate the location of the square. This was repeated until participants made two errors or fewer. In the practice phase, No-Go trials, where the response must be withheld, were introduced (red squares in the simple task; a blue square following a yellow square in the complex task) (Fig 2 in main text). The practice phase was repeated until participants made no more than one No-Go error out of three No-Go trials. Test phases followed, and included 80 trials, with 25% No-Go trials, and a self-timed break half-way through. The square’s location and fixation duration were pseudo-randomised so that for every set of ten trials, 50% of stimuli appeared on the left, and fixation duration was randomly chosen from a uniform distribution between 600 and 800 ms. Responses were not recorded for the first 100 ms after stimulus presentation because such a quick response would most likely relate to the previous trial. There were never two No-Go trials in a row. Accuracy and RT were recorded, and the two Go/No-Go tasks lasted a total of six min on average.

The numerical Stroop task, adapted from Khng and Lee [2] provided a computerized measure of semantic inhibition (Fig 2 in main text). The aim in this task was to press the key corresponding to the number of elements on the screen (between one and four). In the familiarization phase, the elements were asterisks. The four number keys were located in the centre of the keyboard, labelled with stickers, and participants were instructed to use the index and middle fingers of their left and right hands, such that the fingers corresponded to numbers one to four from left to right. The familiarization phase was repeated until participants responded correctly on 11 out of 12 trials. This conservative threshold ensured that mapping between fingers, keys and responses had been achieved. In the first practice phase, consisting of 24 trials, the stimuli were single digit numbers, and participants continued to respond to the number of elements (digits) shown. These were all congruent trials, where the digit matched the number of elements (e.g., “1” or “3 3 3”). The second practice phase contained only incongruent trials, where the digit did not match the number of elements (e.g., “4 4” or “1 1 1 1”). The second practice contained just two trials, and was repeated until participants got both correct. This ensured that participants understood the task but did not get too much practice.

The test phase contained 50% congruent and 50% incongruent trials presented in four blocks of 24 trials. The same pseudo-random trial order was used across participants, such that each block contained one of each possible incongruent trial and three of each congruent trial. There was a self-timed break between each block. Accuracy and RT were recorded, and the task took four and a half min on average. This numerical version of the Stroop task allowed a more intuitive mapping between numbers and keys as compared to non-ordinal stimuli, such as colours, in the traditional colour-word Stroop task.

**Statistical analysis**

Mean RTs of the inhibitory control tasks are reported for correct trials only. The simple and complex Go/No-Go tasks were analysed separately. Three participants were excluded from the simple Go/No-Go analysis because of low accuracy (one 12y, one 13y) or high RT (one 12y) leaving a final *N*=87 participants. Two participants were excluded from the complex Go/No-Go analysis, because of low accuracy (one 12y) or an inability to pass the practice (one 12y) leaving a final *N*=88 participants. Four participants were excluded from the Stroop task because of low accuracy (one 12y, one 13y) or because they were unable to perform the task due to a hand injury (one 13y, one 15y) leaving a final *N*=86 participants. Two (Trial type: Go, No-Go or congruent, incongruent) x four (Age group: 12y, 13y, 14y, 15y) mixed model repeated measures ANOVAs were run on accuracy scores in each of the three tasks and on RT in the Stroop task. One-way ANOVAs examined the effect of Age group (12y, 13y, 14y, 15y) on Go RT in the simple and complex Go/No-Go tasks separately.

**Supplementary results**

**Age results for accuracy in the science and maths misconception task**

There was a main effect of Age group on accuracy, *F*(3, 83) = 5.61, *p* = .001, *η*_p_^2^ = .169. Follow-up planned comparisons revealed significant differences between 12y and 15y, *p* < .001, 13y and 15y, *p* = .002, and marginal differences between 14y and 15y, *p* = .077, each of which demonstrated increasing accuracy with age (see Table 2 in main text).

There was a significant interaction between Discipline and Age group *F*(3, 83) = 3.68, *p* = .015, *η*_p_^2^ = .117. Follow-up repeated measures ANOVAs performed separately in each Discipline showed a significant effect of Age group for science trials *F*(3, 83) = 4.95, *p* = .003, *η*_p_^2^ = .152 and maths trials *F*(3, 83) = 5.15, *p* = .003, *η*_p_^2^ = .157. Bonferroni-corrected post-hoc comparisons (Table A) revealed significant increases in accuracy between 12y and 15y in science, *p* = .043, and maths, *p* = .002, and between 13y and 15y in science, *p* = .002.

**Table A. Accuracy estimated marginal means in science and maths trials by age group.**

|  | **Accuracy (%)** | |
| --- | --- | --- |
|  | **Science** | **Maths** |
| **Age Group** | ***M (SE)*** | ***M (SE)*** |
| 12y | 66 (1.8)^a^ | 65 (1.3)^b^ |
| 13y | 64 (1.8)^b^ | 69 (1.4) |
| 14y | 69 (1.9) | 69 (1.4) |
| 15y | 73 (2.0) | 72 (1.5) |

^a^ *p* < .05, ^b^ *p* < .01 in comparison with 15y group.

**Inhibitory control tasks**

Mixed repeated measures ANOVAs performed on accuracy in the Go/No-Go tasks revealed a main effect of Trial type for both the simple, *F*(1, 83) = 93.37, *p* < .001, *η*_p_^2^ = .529, and complex tasks, *F*(1, 84) = 183.31, *p* < .001, *η*_p_^2^ = .686, with higher accuracy for Go trials than No-Go trials (Table 3 in main text). On average, 53% of Go errors were omissions. There was a main effect of Age group on accuracy for the simple Go/No-Go task only, *F*(3, 83) = 3.16, *p* = .029, *η*_p_^2^ = .102 (complex *p* = .725), and planned post-hoc comparisons revealed a significant difference between 12y and 15y, *p* = .029 (other *p*s < .70).

Similarly, one-way ANOVAs revealed a marginal effect of Age group on RT in the simple task only, *F*(3, 83) = 2.36, *p* = .078 (complex *p* = .530). Planned post-hoc comparisons showed a significant difference between 12y and 15y, *p* = .020, and between 13y and 15y, *p* = .023, but only marginally between 14y and 15y, *p* = .090. In all cases, RTs were faster in the older age group (Table 3 in main text).

In the numerical Stroop task, the repeated measures ANOVAs revealed a significant main effect of Trial type for both accuracy, *F*(1, 82) = 224.29, *p* < .001, *η*_p_^2^ = .732, and RT, *F*(1, 82) = 426.67, *p* < .001, *η*_p_^2^ = .839, with greater accuracy and faster RTs for congruent trials than incongruent trials (Table 3 in main text). There was also a main effect of Age group in accuracy, *F*(3, 82) = 3.58, *p* = .017, *η*_p_^2^ = .116, but not RT, *p* = .122. Post-hoc comparisons showed significantly poorer accuracy at 12y than 15y only, *p* = .013 (all other *p’*s > .92) (Table 3 in main text). The interaction between Trial type and Age group was not significant (*p* = .622).

In summary, No-Go errors (errors of commission) were more common than Go errors (errors of omission and side judgement) in both the simple and the complex Go/No-Go tasks. Accuracy in the Stroop task was higher and correct responses faster in congruent trials, where the number matched the quantity. There were age effects for accuracy and RT on the simple Go/No-Go task, and accuracy in the Stroop, with better performance in the oldest age group compared to the youngest age groups.

**Correlation analyses between tasks**

Correlations between the variables of interest were examined (Table B) to test assumptions regarding multicollinearity for the main regression analyses.

**Table B. Pearson Correlation Coefficients of Regression Variables for Science and Maths Combined.**

| **Variable** | **1** | **2** | **3** | **4** | **5** | **6** | **7** | **8** | **9** | **10** | **11** | **12** | **13** | **14** | **15** | **16** |
| --- | --- | --- | --- | --- | --- | --- | --- | --- | --- | --- | --- | --- | --- | --- | --- | --- |
| Science and maths |  |  |  |  |  |  |  |  |  |  |  |  |  |  |  |  |
| 1. Misconception accuracy |  |  |  |  |  |  |  |  |  |  |  |  |  |  |  |  |
| 2. Misconception RT | .04 |  |  |  |  |  |  |  |  |  |  |  |  |  |  |  |
| 3. Control accuracy | **.39^c^** | .22 |  |  |  |  |  |  |  |  |  |  |  |  |  |  |
| 4. Control RT | -.01 | **.89^c^** | .05 |  |  |  |  |  |  |  |  |  |  |  |  |  |
| Control variables |  |  |  |  |  |  |  |  |  |  |  |  |  |  |  |  |
| 5. Age (months) | **.38^c^** | -.11 | **.27^a^** | -.16 |  |  |  |  |  |  |  |  |  |  |  |  |
| 6. WASI Vocabulary raw | **.31^b^** | -.04 | **.51^c^** | -.14 | **.21^a^** |  |  |  |  |  |  |  |  |  |  |  |
| 7. WASI Matrix Reasoning raw | .13 | **.21^a^** | .21 | .14 | -.02 | .15 |  |  |  |  |  |  |  |  |  |  |
| Go/No-Go |  |  |  |  |  |  |  |  |  |  |  |  |  |  |  |  |
| 8. Simple No-Go accuracy | .17 | **-.24^a^** | .02 | -.18 | .21 | -.01 | -.20 |  |  |  |  |  |  |  |  |  |
| 9. Complex No-Go accuracy | .03 | .09 | .07 | .01 | -.04 | .09 | -.09 | .18 |  |  |  |  |  |  |  |  |
| 10. Simple Go accuracy | .13 | -.14 | .14 | -.12 | .15 | .06 | .00 | **.48^c^** | .10 |  |  |  |  |  |  |  |
| 11. Complex Go accuracy | **.24^a^** | -.04 | .12 | -.08 | .18 | .06 | .13 | .00 | -.09 | .10 |  |  |  |  |  |  |
| 12. Simple Go RT | -.13 | **.24^a^** | **-.24^a^** | **.35^b^** | **-.33^b^** | -.16 | **-.22^a^** | .11 | .07 | -.19 | **-.21^a^** |  |  |  |  |  |
| 13. Complex Go RT | -.04 | **.22^a^** | -.10 | **.31^b^** | -.07 | -.15 | -.10 | **.28^b^** | .13 | .07 | **-.23^a^** | **.66^c^** |  |  |  |  |
| Numerical Stroop |  |  |  |  |  |  |  |  |  |  |  |  |  |  |  |  |
| 14. Accuracy cost | .03 | -.03 | -.04 | -.05 | -.04 | -.12 | .06 | **-.25^a^** | -.19 | -.03 | .13 | **.28^b^** | -.21 |  |  |  |
| 15. RT cost | -.16 | .06 | -.05 | .09 | .06 | -.09 | -.05 | .14 | -.18 | -.05 | -.07 | .15 | .06 | -.11 |  |  |
| 16. Congruent accuracy | .20 | -.10 | **.23^a^** | -.08 | **.42^c^** | .05 | -.07 | **.48^c^** | .10 | **.36^c^** | -.01 | .11 | **.24^a^** | **-.27^a^** | **-.29^b^** |  |
| 17. Congruent RT | -.06 | .14 | -.14 | **.22^a^** | **-.23^a^** | -.15 | -.13 | -.02 | -.04 | **-.23^a^** | -.12 | .**56^c^** | **.44^c^** | **-.26^a^** | .01 | .05 |

Statistically significant (two-tailed) correlations are highlighted in bold, ^a,b,c^ indicate *p* <.05, *p* < .01 and *p* < .001 respectively.

**References**

1. Watanabe J, Sugiura M, Sato K, Sato Y, Maeda Y, Matsue Y, et al. The human prefrontal and parietal association cortices are involved in No-Go performances: An event-related fMRI study. Neuroimage. 2002;17(3): 1207-1216. doi: 10.1006/nimg.2002.1198.
2. Khng KH, Lee K. The relationship between Stroop and stop-signal measures of inhibition in adolescents: Influences from variations in context and measure estimation. PLoS One. 2014;9(7): e101356. doi: 10.1371/journal.pone.0101356.
